# Risk Measurement of Perinatal and Neonatal Morbidity Characteristics and Applicability of GAIA Case Definitions: Results and Lessons Learnt of a Hospital-Based Prospective Cohort Study in the Valencia Region (2019–2020)

**DOI:** 10.3390/ijerph19127132

**Published:** 2022-06-10

**Authors:** Antonio Carmona, María Latorre Tejerina, Alicia Martínez Sebastián, Dafina Dobreva, Cristina P. Jurca, Sergio Huerta Barberá, Vicente Bernat Montoya, Mercedes Aristoy Zabaleta, Ana Pineda Caplliure, Beatriz Mansilla Roig, María Navío Anaya, Ricardo Tosca-Segura, Miguel Tortajada-Girbés, Javier Díez-Domingo, Alejandro Orrico-Sánchez

**Affiliations:** 1 Vaccines Research Department, Fundación Para el Fomento de la Investigación Sanitaria y Biomédica de la Comunitat Valenciana (Fisabio), Av. de Catalunya, 21, 46020 Valencia, Spain; javier.diez@fisabio.es (J.D.-D.); alejandro.orrico@fisabio.es (A.O.-S.); 2 Department of Pediatrics, Hospital General Universitario de Castellón, Avinguda de Benicàssim, 128, 12004 Castelló de la Plana, Spain; mlatorretejerina@gmail.com (M.L.T.); cristinapaulajurca.med@gmail.com (C.P.J.); sergiohb93@gmail.com (S.H.B.); vibermon3@hotmail.com (V.B.M.); merxita92@gmail.com (M.A.Z.); tosca_ric@gva.es (R.T.-S.); 3 Department of Pediatrics, Hospital Universitario Doctor Peset, Av. de Gaspar Aguilar, 90, 46017 Valencia, Spain; aliciamarsebastian@gmail.com (A.M.S.); anapicap@gmail.com (A.P.C.); bemaro1402@gmail.com (B.M.R.); mnavioanaya@gmail.com (M.N.A.); tortajadamig@gmail.com (M.T.-G.); 4 P95 Pharmacovigilance and Epidemiology, 3010 Leuven, Belgium; dafina.dobreva@p-95.com; 5 Universidad Católica de Valencia San Vicente Mártir, Carrer de Quevedo, 2, 46001 Valencia, Spain

**Keywords:** vaccination, pregnancy, maternal immunization, vaccine safety, standardized case definitions, incidence rates, neonatal outcomes

## Abstract

Post-marketing safety surveillance of new vaccines aimed to be administered during pregnancy is crucial to orchestrate efficient adverse events evaluation. This is of special relevance in the current landscape of new vaccines being introduced in the pregnant women population, and particularly due to the recent administration of COVID-19 vaccines in pregnant women. This multi-center prospective cohort study, nested within the WHO-Global Vaccine Safety-MCC study, involved two hospitals in the Valencia region. Hereby, the incidence rates of seven perinatal and neonatal outcomes in the Valencia region are presented. The pooled data analysis of the two Valencian hospitals allowed the estimation of incidence rates in the Valencia Region (per 1000 live births): 86.7 for low birth weight, 78.2 for preterm birth, 58.8 for small for gestational age, 13 for congenital microcephaly, 0.4 for stillbirth, 1.2 for neonatal death and 6.5 for neonatal infection. These figures are in line with what is expected from a high-income country and the previously reported rates for Spain and Europe, except for the significantly increased rate for congenital microcephaly. Regarding the data for maternal immunization, the vaccination status was collected for 94.4% of the screened pregnant women, highlighting the high quality of the Valencian Vaccine Registry. The study also assessed the Valencian hospitals’ capacity for identifying and collecting data on maternal immunization status, as well as the applicability of the GAIA definitions to the identified outcomes.

## 1. Introduction

Through 2019, neonatal death accounted for approximately 47% of the mortality among children less than 5 years of age globally and of these deaths, 11% were vaccine-preventable deaths caused by infections [1]. In this context, new vaccines are being developed to be administered during pregnancy to protect neonates from infection, such as vaccines against the Zika virus, respiratory syncytial virus (RSV), and hepatitis E [2]. Furthermore, as a consequence of the COVID-19 pandemic, the vaccines developed against the SARS-CoV-2 have been extensively used in pregnant women.

One of the primary concerns of vaccination during pregnancy is the safety of the vaccines. The benefit-risk of vaccines in pregnancy has to be assessed by balancing vaccine safety with the risk of infection for the pregnant woman and her fetus or infant in the absence of immunization [3]. This requires the assessment of rates of adverse events following immunization (AEFI) that have been previously reported for large populations [3,4]. Vaccine safety surveillance is complex, and even more so during pregnancy, as it is difficult to separate adverse pregnancy outcomes that are due to exposures to infections/vaccination from those caused by other factors. This requires a strong safety surveillance system able to collect detailed, unbiased, and accurate data, which is even more challenging in low- and medium-income countries (LMICs) where there is a lack of baseline data and adequate pharmacovigilance infrastructure [5].

Therefore, post-marketing safety surveillance of new vaccines, which will be administered during pregnancy, is an important mechanism to evaluate adverse events in pregnant women and neonates/infants [5,6]. This surveillance can be of benefit as it will inform the setting of background rates for various health outcomes that are not available for various LMICs, especially in the current landscape of new vaccines being introduced in the pregnant women population.

In response to the call of WHO for a globally concerted approach to monitoring the safety of vaccines in pregnancy, the Global Alignment of Immunization Safety Assessment in Pregnancy (GAIA) project was launched in 2015 [2,7,8] with the objective of establishing standardized definitions for obstetric and neonatal outcomes to increase the comparability of safety data across research studies and surveillance systems. In the present study, we will also assess the applicability of GAIA case definitions to our recruited cases, in order to inform future vaccine safety studies and provide a gold-standard reference for the implementation of these definitions in the LMIC countries as part of the WHO-GVS-MCC study [6,7,8,9,10].

This multi-center prospective descriptive cohort study, nested within the WHO-GVS-MCC study [9,10,11], involved two hospitals from the Valencia region. Spain was the only high-income country represented in the WHO-GVS-MCC study, serving as a reference to LMICs in terms of risk measurement of early childhood outcomes and applicability of specific neonatal outcomes case definitions.

The study was conducted using routinely collected data at two Valencian hospitals, and the information on seven perinatal and neonatal outcomes (low birth weight, preterm birth, small for gestational age, stillbirth, congenital microcephaly, neonatal death, and neonatal infection) was recorded for all the newborns. Moreover, maternal immunization status was also collected.

This piece of work presents the incidence rates of several maternal, perinatal, and neonatal outcomes in the Valencia region, with the aim of estimating the minimum detectable risk of these outcomes using standardized case definitions. The study also assessed the Valencian hospitals’ capacity for identifying and collecting data on maternal immunization status, as well as the applicability of the GAIA definitions to the identified outcomes.

## 2. Materials and Methods

### 2.1. Study Setting

**Figure 1:**
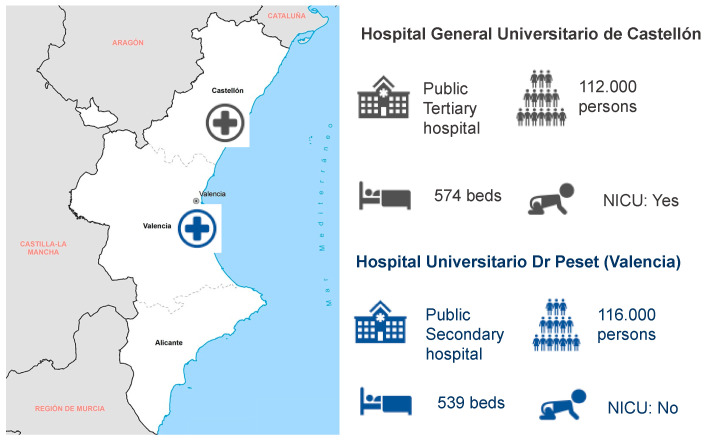
Geographic situation and characteristics of the two participant hospitals in the Valencia Region (Hospital General de Castellón and Hospital Universitario Dr. Peset): facility ownership, catchment population, number of beds, and presence of a neonate intensive care unit (NICU).

In the Valencia Region (Spain), two hospitals collected data for the study: Hospital General Universitario de Castellón (tertiary hospital in the Castellon province) and Hospital Universitario Dr. Peset (secondary hospital in the Valencia province) (Figure 1).

### 2.2. Study Design

A prospective descriptive cohort study using routinely collected data at two hospitals in the Valencia Region in Spain was conducted. The generic study protocol was developed by WHO and adapted by FISABIO to the local recruitment, data collection, and setting conditions.

### 2.3. Study Outcomes

The outcomes were selected based on relevance and perceived complexity of data collection. The variables of interest for this study were defined as follows [9,10]:

- *Low birth weight (LBW):* first weight of <2500 g recorded in the birth register or patient record.
- *Preterm birth (PTB):* reported gestational age (GA) of < 37 weeks.
- *Small for gestational age (SGA):* diagnosis of small for gestational age or small for date recorded in the patient records.
- *Stillbirth (SB):* fetal death occurring before birth after a selected, predefined duration of gestation, recorded in the patient records. Both intrapartum stillbirth and antepartum stillbirth are considered. The predefined duration of gestation varies between 22 and 28 weeks across sites.
- *Congenital microcephaly (CM):* postnatal diagnosis of congenital microcephaly (live births only) recorded in the patient records. The diagnostic charts used are the OMS charts.
- *Neonatal death (ND):* death of a live-born child within 28 days of birth recorded in the patient record.
- *Neonatal infection (NI):* diagnosis of an invasive bloodstream infection (BSI), respiratory infection, or meningitis within 28 days of birth recorded in the patient records.
- *Exposure to maternal immunization (MI)* was defined as any indication of receipt of any vaccination during pregnancy.

### 2.4. Study Population

The study population consisted of all infants that met the inclusion criteria below:

- Born at the site (including stillborn) within 12 months of study start,
- Diagnosed at the site with one of the study outcomes at birth or within 28 days of birth.
- Informed consent from the mother.

In order to assess maternal immunization, corresponding mothers to the infants recruited were also part of the study population.

### 2.5. Study Period

The study started in May 2019 and finished in July 2020. Outcomes occurring within 28 days of birth were prospectively identified among infants born over a 12-month period.

### 2.6. Recording Births and Screening for Outcomes

Births were typically ascertained through the birth register in the labor ward. All births were recorded in the electronic data capture system for a duration of 12 months from study start. No individual-level data could be recorded without informed consent, therefore only the total aggregated number of births per month was recorded.

Outcomes were generally identified by manual review of electronic registers, such as birth and admission registers, and patient records from maternity, neonatal or pediatric wards, and from neonatal intensive care units (NICUs). Only sources at the site were screened.

Relevant source documents were screened to identify and record the presence of CM, LBW, ND, NI, PTB, SGA, and SB, within 28 days of birth, among neonates born at the site. There was no active follow-up to identify outcomes of interest that may have occurred outside the sites following discharge of the newborn from the hospital.

Up to 100 cases per outcome were systematically recruited at each site [9,10]. For each recruited case, case report forms (CRFs) were completed from a variety of sources, including the mother’s antenatal care records, the antenatal care card, and patient records. Background information, including receipt of any vaccines by the mother during pregnancy or within 30 days prior to the last menstrual period (LMP), and outcome-specific information was collected.

### 2.7. Valencian Vaccine Registry

The vaccination status was obtained from The Valencia healthcare Integrated Databases (VID) [12]. The Vaccine Information System (VIS) provides vaccination status and information about all vaccine doses administered both in public and many private healthcare centers. The data included are the type of vaccine, the batch number, the number of dose/s, the place and date of administration, and when applicable, if the individual is part of a risk group.

### 2.8. Statistical Analysis

The incidence rates were calculated as the total number of specific outcomes identified during the study period divided by the total number of live births during the study period, times 1000. The 95% confidence intervals were calculated using the exact Clopper-Pearson method.

Statistical analysis at site-specific level was performed by P95 as part of the WHO-GVS-MCC study. The statistical analysis for Valencia region overall was performed by FISABIO.

## 3. Results

### 3.1. Descriptive Analysis

A total of 2468 births were registered in the defined study period, of which 1390 were recorded in Castellon GUH, whereas 1078 were recorded in the Dr. Peset UH (Table 1). Moreover, a total of 604 perinatal and neonatal outcomes were identified: 411 in Castellon GUH and 193 in Dr. Peset UH (Table 2).

From the 604 total cases identified, 35.4% and 32% were LBW and PB, respectively. The 26.1% of the observed cases (106 in Castellón GUH, 52 in Dr. Peset UH for a total of 158 total cases) presented more than one outcome simultaneously. The most often used combinations were LBW and PTB, LBW and SGA or PTB, and LBW and SGA together.

The most commonly identified perinatal/neonatal outcome was LBW, with a total of 214 cases identified in both hospitals, followed by PTB, registered in 193 cases and SGA, with 145 cases (Table 2). A total of 32 cases of CM were identified, and only three cases of ND, and 1 SB were registered.

Neonatal infections were classified into three different categories: respiratory infections, invasive bloodstream infections and meningitis. Among the 16 neonatal infection cases identified (diagnosed up to 28 days after birth), 14 (87.5%) were registered as invasive bloodstream infection, one case (6.25%) as neonatal meningitis, and another case of respiratory infection (6.25%).

### 3.2. Estimation of Incidence Rates of Neonatal and Perinatal Outcomes in the Valencia Region

**Figure 2:**
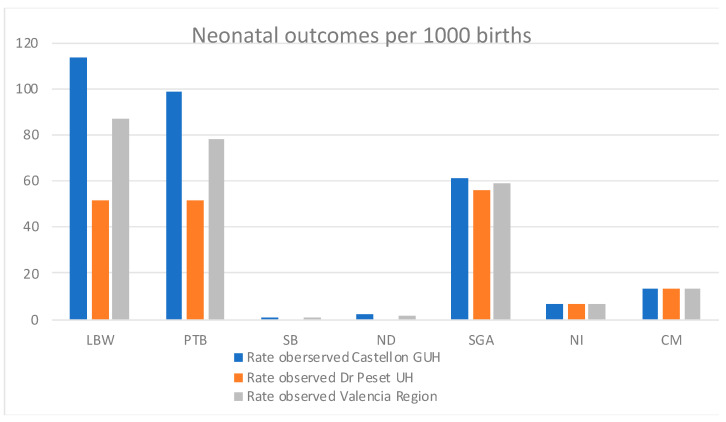
Incidence rates (per 1000 live births) for the different perinatal and neonatal outcomes identified, per study site and overall for the Valencia Region.

The rates for the different perinatal and neonatal outcomes were estimated for both study sites and overall, for the Valencia Region (Table 3, Figure 2).

Incidence rates of LBW, PTB, and SGA neonatal outcomes were 86.7, 78.2, and 58.8 per 1000 live births overall in the Valencia Region (Table 3, Figure 2). Previously reported incidence rates in Spain were 83 [13] and 70 [14] per 1000 live births for LBW and PTB, respectively. Moreover, reported rates in Europe overall were 65 (UN), 73 (WHO), and 79 (UNICEF) per 1000 live births for LBW [13]; and 87 per 1000 live births for PTB [14]. Previously reported rates of SGA in Spain specifically were not found in the literature, but in Europe, the SGA incidence rate was 64 per 1000 live births [15]

The incidence rate of CM in the Valencia Region was 13 per 1000 live births (Table 3, Figure 2)., which contrasts with the previously reported incidence rate in Spain, which was 0.29 per 1000 live births in 2012 [16], and 0.15 per 1000 live births in Europe overall [16].

Only one stillbirth and three neonatal deaths were recorded in the Valencian study for the defined study period (all at Castellon GUH), being the overall incidence rates 0.4 and 1.2 per 1000 live births, respectively (Table 3, Figure 2). The previously reported SB rate for Spain was 2.2 in 2019 [17] and 2.4 per 1000 live births in 2015 [18]. Similarly, the SB incidence rate in Europe, North America, Australia, and New Zealand overall (in 2019) was 3.1 per 1000 live births in 2019 [17], and for Europe only (in 2015) was 2.9 per 1000 live births [18]. On the other hand, the ND incidence rate reported in 2019 in Spain was 2 per 1000 live births, whereas in Europe overall, 3 per 1000 live births [19].

Finally, with respect to the incidence rate for neonatal infections in the Valencia region, a 6.5 per 1000 birth rate was estimated for the total number of neonatal infections, whereas 5.7, 0.4, and 0.4 per 1000 births were estimated for invasive bloodstream infection, meningitis and respiratory infection, respectively (Table 3, Figure 2).

### 3.3. Maternal Immunization Status Ascertainment in the Valencia Region

A total of 247 mothers of the recruited neonates were subsequently recruited for the WHO-GVS-MCC study in Valencia. The percentage of vaccinated mothers among the recruited ranged from 85.7% (Castellón GUH) to 88.4% (Dr. Peset UH). Overall, in the Valencia Region, the percentage of vaccinated mothers was 86.6% (Table 4). A 7.3% of the mothers were unvaccinated, whereas the vaccination status of 15 mothers was unknown (6.1%), as the information was missing from the Valencian Vaccine Registry.

For the Valencian study, the target disease of the vaccination was always known (100%). The majority of vaccine doses were administered during the third trimester (50.7%), and for 31% of the doses, the time of vaccination was unknown (Table 5). Most vaccinations were against pertussis (or pertussis in combination with other vaccines) and influenza.

### 3.4. Applicability of GAIA Definitions to Neonatal Outcomes

From the 611 neonatal outcomes identified during the study period, only 445 were successfully recruited to the WHO-GVS-MCC study and considered for the GAIA definitions applicability assessment (284 in Castellon GUH, 161 in Dr. Peset UH) (Table 6).

Castellón GUH was able to recruit 69% of the cases screened, whereas Dr. Peset UH recruited 83.4% of the cases screened from all the live births (Table 6).

**Figure 3:**
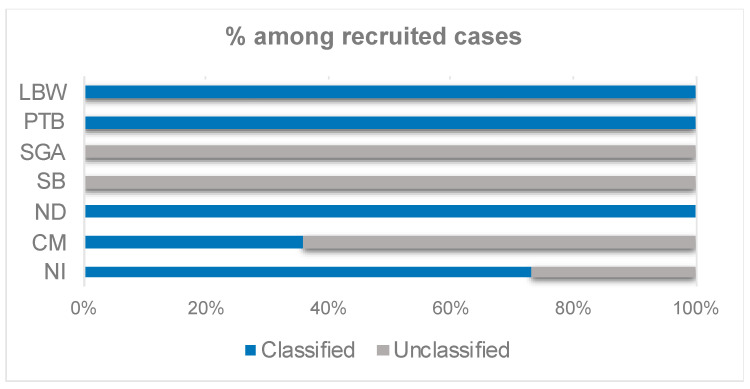
Percentage of classification of the cases recruited in Valencia into any of the diagnostic certainty levels of the GAIA definitions, per neonatal outcome of interest.

The totality of the LBW (100%, 151 cases) and PTB recruited cases (100%, 145 cases) were classified into any of the levels of GAIA diagnostic certainty (Figure 3). In addition, 105 cases of SGA were recruited at both hospitals, but none of them could be classified in any of the GAIA diagnostic certainty levels for SGA (Figure 3), as the calibration of the balance was considered unknown.

There was one stillbirth case recruited at Castellón GUH. However, it was not classified among any of the GAIA diagnostic certainty levels of the stillbirth outcome (Figure 3). The reason was that there was insufficient information to distinguish between antepartum and intrapartum stillbirth, as there was no record of signs of life prior to the onset of labor and there was very little information on gestational age in that particular case.

A total of twenty-five congenital microcephaly cases were recruited at the Valencian hospitals but just nine cases (36% of the recruited cases) were classified in any of the GAIA levels of diagnostic certainty for the microcephaly outcome (Figure 3, Supplementary Table S1). Three neonatal death cases were recruited in Castellón GUH, and no cases were registered or recruited for Dr. Peset UH. All of the three recruited cases in Castellon were classified in the highest level of diagnostic certainty, as per GAIA definitions (Figure 3). Finally, fifteen cases of neonatal infection were recruited in the two participating Valencian hospitals, being four of the recruited cases (26.7%) were unclassified (Figure 3, Supplementary Table S3).

### 3.5. Applicability of GAIA Definitions to Maternal Immunization

Maternal immunization was assessed retrospectively (as mothers were recruited into the study after giving birth) and the ascertainment of the vaccination status was based on the information present in the Valencian Vaccine Registry. Thus, mothers were not asked about their immunization status during pregnancy solely for the purpose of this study. In the participating hospitals of the Valencia region, the vaccination status of the mother was known in 93.9% of the recruited cases. From all the mothers with a known vaccination status, 86.7% were vaccinated (Table 7).

**Figure 4:**
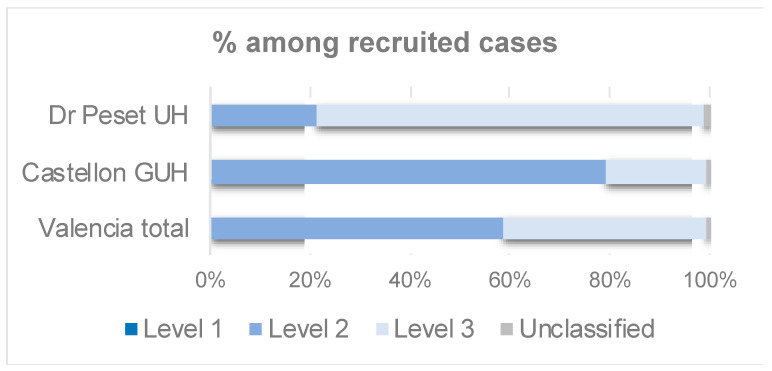
Maternal immunization: Bar chart showing the percentage of the vaccinated mothers from recruited cases classified by GAIA level of diagnostic certainty.

A 59% of the vaccinated mothers classified into any diagnostic certainty level were classified into level 2, whereas a 41% were assigned to the level 3 definition (Table 7, Figure 4). None of the vaccinated cases was classified as level 1, due to unavailability of the time of immunization in the Valencian Vaccine registry (Table 7). The maternal immunization definitions for the three GAIA levels of diagnostic certainty can be found in the Supplementary Methods.

Furthermore, in Dr. Peset UH, in the 100%, 98% and 88% of the cases defined as level 3, the vaccination date, vaccine brand, and batch number were not available, respectively (Supplementary Table S4). On the other hand, the information on the specific diseases targeted for the vaccination was generally available and only one mother with a date of immunization that was not during the pregnancy was identified, in Castellon GUH (Supplementary Table S4).

## 4. Discussion

### 4.1. Estimation of Incidence Rates of Neonatal and Perinatal Outcomes in the Valencia Region

The occurrence rate of seven perinatal and neonatal outcomes in the Valencia Region has been estimated, building from the results obtained as a part of the WHO-GVS-MCC global study [10]. Interestingly, all of the previous outcomes were identified during the first week of the life of the newborn.

Overall, more than double the outcomes of interest were recruited in Castellón GUH (411), in comparison to Dr. Peset UH (193). This is explained by the nature of the Castellón GUH hospital, which is a reference tertiary hospital. In contrast to Dr. Peset UH, which is a secondary hospital, Castellón GUH hospital has a neonatal intensive care unit. As a consequence, although the catchment population of both hospitals is similar, Castellon GUH also receives the potential preterm neonates corresponding to other secondary hospitals in the Castellon province. This is not the case for the Dr. Peset hospital, which only admits preterm births of >32 weeks of gestation, thus outcomes such as SGA, PTB, or ND are more unlikely.

**Figure 5:**
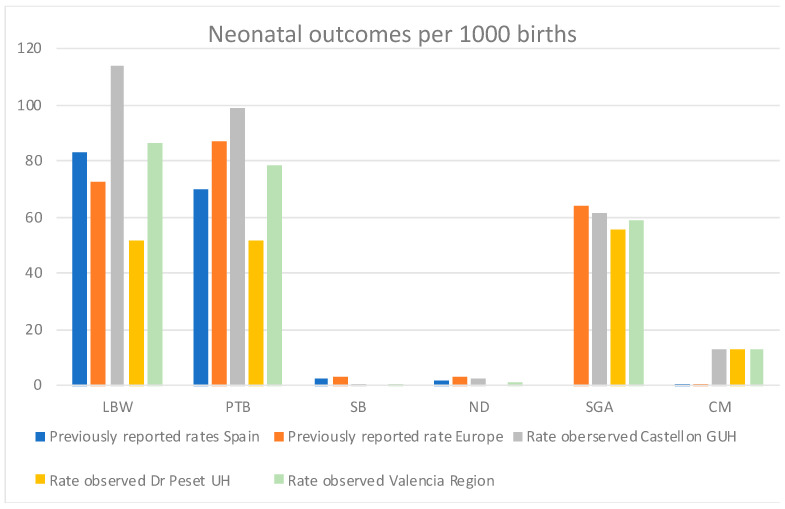
Comparative on the incidence rates of neonatal outcomes estimated in the Valencian study versus rates reported in previous literature for Spain and Europe overall. Previously reported rates for SGA in Spain are not available. Previously reported rate of LBW for Europe is an average of the rates reported by the UN, WHO, and UNICEF [13].

The data analysis performed in the present study yielded incidence rates of the outcomes of interest for the Valencia region (Figure 2) that are in line with the previously reported rates in Spain and Europe for the LBW, PTB, SB, and ND outcomes ([13,14,17,18,19], Figure 5). The rates for SGA are also resembling the rates reported for Europe [15], but we could not find any literature reporting the specific rates for Spain.

Strikingly, in contrast with the previous literature, we observe a higher microcephaly incidence rate (Figure 5). The incidence rate of congenital microcephaly in the Valencia Region was 13 per 1000 live births at both sites, whereas the incidence of congenital microcephaly previously reported were 0.15 and 0.29 per 1000 live births in Europe and Spain [16], respectively. The determination of congenital microcephaly is highly dependent on the measuring procedures and the training of the staff at each hospital. Most of the neonatal microcephalies identified are unlikely to be congenital microcephalies (e.g., caused by cytomegalovirus or Zika infection in the mother), but temporary microcephalies produced by cranial molding at birth or other reasons. As a matter of fact, the literature reports that the measurement of the cranial perimeter (CP) is prone to errors, thus the recommendation of measuring at least three times for confirmation of microcephaly [20].

In Castellón GUH and Dr. Peset UH, the cranial perimeter was measured and registered only once by the nurses (not the pediatricians participating in the study), regardless of the percentile corresponding to the measurement. The lack of a second and third measurement by the pediatricians and the tendency for errors in the CP measurement might be the reason why such a high incidence rate for CM is observed. The doctors reported that most of the cranial perimeter anomalies observed were barely meeting the microcephaly percentile criteria and therefore it is highly unlikely that all of them are real congenital microcephalies. Thus, the incidence rates presented in the results have to be interpreted with caution, as these are probably overestimated.

In Dr. Peset UH, all the mothers of the suspected microcephaly cases are taken a urine sample and tested in the lab for cytomegalovirus, as this is the most frequent reason for congenital microcephaly [21,22]. The doctors did not identify any positive cytomegalovirus diagnosis for the CM cases reported in the present study, supporting the hypothesis of measurement errors as the main reason for the higher CM incidence rates.

Finally, it was challenging to find consistent and comparable rates for neonatal infections in Europe and Spain in the existing literature. Thus, we did not provide a comparison for the neonatal infection outcome in Figure 5. Nevertheless, in comparison with other LMICs also participating in the WHO-GVS-MCC study [10], the Valencian rates were considerably lower, except for meningitis (Supplementary Figure S1). It is also worth mentioning two points that might have influenced the incidence rates of infection in the present study:

- neonatal infections were not followed-up outside of the facility, therefore, these rates are likely lower than the true rates reported in the present study.
- currently, the general procedure, even in the cases of infection suspicion (e.g., after a prolonged premature membrane break), is to not test for microbiological confirmation of the infection, but to monitor the evolution of infection and the neonate constants. Thus, it is likely that some cases of infection have not been reported as such as they did not have a lab confirmation, underestimating the incidence rates reported.

The interpretation of the estimated incidence rates in the Valencia Region should be done with caution, as Castellón GUH is a tertiary referral hospital to which all complicated pregnancies are referred and may show higher rates than the national average reported. Moreover, the identification of SGA, and CM outcomes was not always systematic and standardized, so it is likely that some cases have been missed during the study period, therefore affecting the accuracy of the incidence rates. However, the overall estimations for the two hospitals are still informative and representative of the Valencia Region.

The estimation of the incidence rates of these neonatal outcomes in the Valencia Region is crucial for the post-authorization safety assessment of newly introduced vaccines. This is currently of relevance, as several new vaccines have been recently introduced (e.g., Zika or COVID-19) or will be soon introduced (e.g., RSV) in the immunization schedule of the pregnant women population.

### 4.2. Applicability of GAIA Definitions to Neonatal Outcomes

From the total number of outcomes screened, Castellón GUH was able to recruit 69% of the cases and Dr. Peset UH recruited 83.4% of the cases. The most common reason for exclusion from the study was the parents refusing to sign the informed consent, especially for those neonates presenting severe outcomes, which were more frequent at Castellón GUH. This was particularly difficult for those neonates admitted to the NICU in the case of the Castellón GUH, which explains the lower percentage of recruitment at the Castellon site. Dr. Peset UH also reported better access to the parents and therefore a swifter informed consent form signature process.

The present results highlighted the high applicability of several of the GAIA definitions to the neonatal outcomes recruited in the Valencian hospitals. The results showed that the case definitions for PTB, LBW, ND, and NI were generally applicable for all the recruited cases, whereas those for SB, CM, and SGA had limited applicability (Figure 3). These findings are in line with the results obtained in the global WHO-GVS-MCC study [9,10].

Even though the applicability of the GAIA definition for NI was high (73.3%-Figure 3), four of the recruited NI cases were not classified into any of the GAIA diagnostic certainty levels as the lab confirmation of the diagnosis was missing. The reason behind this is that the standard hospital procedure, even in the cases of infection suspicion (e.g., after a prolonged premature membrane break), is to not test at the microbiology lab, but to monitor the evolution of infection and the neonate constants.

None of the SGA cases could be classified in any of the GAIA diagnostic certainty levels for SGA (Figure 3). The main reason for the misclassification was that the frequency of the balance calibration was unknown, therefore, the GAIA definition considered that the weight was not obtained using appropriate criteria to be classified as level 3. Despite the doctors not being able to determine when exactly the balance was calibrated, it was confirmed that the balance was regularly calibrated by hospital technicians. Consequently, the SGA GAIA definition was considered too strict and will need to be redefined, as the rest of the criteria in the definition were met in all the SGA cases recruited.

Moreover, insufficient information to distinguish between antepartum and intrapartum stillbirth, as there was no record of signs of life prior to the onset of labor and there was very little information on gestational age in that particular case.

With respect to CM, 64% of the recruited cases were not classified into any of the GAIA levels of diagnostic certainty (Figure 3, Supplementary Table S1). The most frequent reasons for not meeting the GAIA definition criteria (therefore, unclassified) were that microcephaly was not diagnosed based on the ICD code algorithm (in the 100% of unclassified cases) and that head circumference was not 2SD below mean or the third percentile according to GA and gender on Intergrowth-21 chart (in 88.2% of the unclassified cases) (Supplementary Table S2).

The use of GAIA case definitions might be challenging in LMIC, as the proposed criteria for some definitions may exceed clinical capacity at some sites [9,10]. The fact that the GAIA definitions had limited applicability in a developed country such as Spain, is a clear indication that the definitions will be more challenging to implement in low-medium income countries, with more reduced infrastructure and pregnancy data availability. Consequently, the study highlighted that the definitions have to be revisited and adjusted for a more efficient application at the site level [11].

### 4.3. Applicability of GAIA Definitions to Maternal Immunization

Regarding the data for maternal immunization, the vaccination exposure information was collected for the 93.9% of pregnant women, emphasizing the high quality of the Valencian Vaccine Registry. Only 6.1% of the mothers had an unknown vaccination status, and those were either immigrants (with less reliable control of the vaccination during pregnancy) or were vaccinated in private hospitals (therefore no record of their vaccination status in the public Valencian Vaccine Registry).

Furthermore, 86.7% of the pregnant women were vaccinated, proving a high vaccination coverage in this particular subpopulation in the region of Valencia, and overall in Spain.

The level 1–2 criteria “immunization recorded in medical records by a health care worker who administered/witnessed the administration of vaccine” was interpreted as primary source medical records, such as the ANC card, vaccine card, or vaccine register. Secondary source medical records, such as the case sheet or the birth recorded, were accepted in level 3.

Several aspects of the definition relating to the vaccine and disease were open to interpretation. For level 1, “date/time” was interpreted as both date and time of administration, and “details of vaccine” as the vaccine lot number, and either the name of the disease or the name of the vaccine. For level 2, “details of the disease” were interpreted as either the name of the disease or the name of the vaccine, or the lot number were required. To ensure standardization between studies, these aspects of the definition should be further specified.

### 4.4. Lessons Learnt

- Improvement of the congenital microcephaly diagnosis: a strikingly higher microcephaly incidence rate was observed in the present study with respect to the rates reported in the previous literature for Europe and Spain. This has been associated with measurement errors, as the diagnosis of microcephaly by measuring the cranial perimeter has been reported to be prone to errors. The present study has highlighted the need to confirm the congenital microcephalies by measuring the cranial perimeter at least three times, and ideally, by different healthcare professionals. Training of the staff in using the WHO reference charts for identifying CM cases will also be essential. The pediatricians at the collaborating hospitals should implement this new procedure and use the lessons learned from the study for a more precise diagnosis of congenital microcephaly.
- Improvement of the Valencian Vaccine Register: even though the Valencian Vaccine Registry is considered one of the top vaccine registries in Europe [12], the vaccine exposure information for several pregnant women was not available (6.1%), with a higher proportion in the Dr. Peset UH (7%). As discussed earlier, this might correspond to immigrant women, whose vaccination is not recorded in the Valencian or any of the regional Spanish vaccine registries (and coincidentally Dr. Peset UH catchment population includes an area with a higher immigrant population) and women attending private medical care outside Spain. Moreover, there is also room for improvement in the way the gynecologists and midwives record the maternal vaccination status in the registry, as its quality is highly dependent on the exhaustiveness of the manual recording made by these doctors and the completeness of the information provided by the mother through the vaccination card or their recollection.
- Interference of COVID-19 pandemic and lockdown in the study: the COVID-19 pandemic started during the study period and interfered with the study conduct since March 2020 [11]. There were no deaths of mothers or neonates due to COVID-19 complications recorded during the study period. Interestingly, the number of screened total births and identified outcomes per month was significantly decreased with respect to the pre-pandemic months, mostly due to the lockdown and the mothers preferring to give birth in private hospitals. This was due to the perceived higher risk of being infected in crowded public hospitals and the lack of staff in the saturated public hospitals as most doctors were tackling the COVID-19 crisis (e.g., anesthetists not being always available to administer the epidural). From July 2019 to March 2020 (pre-pandemic months), there was an average of 227.2 total births and 55.9 outcomes identified per month, whereas from April 2020 to July 2020 there was an average of 170.6 total births and 34 outcomes identified per month. Consequently, the COVID-19 pandemic reduced the number of total births and outcomes identified in the present study, especially in the strictest lockdown months (March to July 2020).

## 5. Conclusions

The present study has provided the incidence rate estimates of seven perinatal and neonatal outcomes and the completeness of the maternal status information in the region of Valencia. The two Valencian hospitals were the only representatives of high-income countries in the WHO-GVS-MCC study, contributing to establishing guidelines for LMIC countries in terms of perinatal and neonatal outcomes identification, registration, and applicability of the GAIA standardized case definitions. Moreover, the study also contributed to the development of expertise in the participating Valencian hospitals and the lessons learned will be implemented in the standard procedures at the respective gynecology and pediatrics departments.

The incidence rates in the Valencia region are in line with the previously reported rates in Spain and Europe for the LBW, PTB, SGA, SB, and ND outcomes. On the other hand, the rates for CM were higher than reported in the previous literature, mainly for errors in the measurement of the cranial perimeter. Although the estimates have to be interpreted with caution, the overall estimations for the two hospitals are still informative and representative of the Valencia Region.

The determination of the incidence rates for these neonatal outcomes sets a precedent in the Valencia Region and paves the way for the post-marketing safety assessment of immunization programs. This is now of special interest, as new vaccines are being introduced in the pregnant women’s immunization schedule, and particularly after the recent administration of COVID-19 vaccines in the pregnant women population.

Finally, the use of GAIA case definitions might be challenging in LMIC, as the proposed criteria for some definitions may exceed clinical capacity at some sites. The fact that the GAIA definitions had limited applicability in a high-income country such as Spain is a clear indication that the definitions will be more complex to implement in LMIC, with more reduced infrastructure and pregnancy data availability. Consequently, the study highlighted that the definitions have to be revisited and adjusted for a more efficient application at the site level.

## Figures and Tables

**Table 1:** Characteristics of participant hospitals and number of total births and live births during the study period. US: Ultrasound.

Site	Type of Healthcare Setting	Catchment Population	Facility Ownership	Presence of NICU	Format of Medical Records	US Facilities	X-ray Facilities	Total Births	Live Births
Castellón GUH	Tertiary	111.162	Public	Yes	Electronic health records	Yes	Yes	1390	1389
Dr. Peset UH	Secondary	116.326	Public	No	Electronic health records	Yes	Yes	1078	1078

**Table 2:** Number of total perinatal and neonatal study outcomes identified at the screening stage during the study period. Number of total cases identified in the study for each of the outcomes, per site and in total in Valencia Region. Abbreviations: ND: neonatal death; SB: stillbirth; LBW: low birth weight; PTB: preterm birth; SGA: small for gestational age; NI: neonatal infection; CM: congenital microcephaly.

Site	Total Outcomes Identified	ND; n (%)	SB; n (%)	LBW; n (%)	PTB; n (%)	SGA; n (%)	NI; n (%)	CM; n (%)
Castellon GUH	411	3 (0.7)	1 (0.2)	158 (38.4)	137 (33.3)	85 (20.7)	9 (2.2)	18 (4.4)
Dr. Peset UH	193	0 (0)	0 (0)	56 (29)	56 (29)	60 (31)	7 (3.6)	14 (7.3)
**Total Valencia**	604	3 (0.5)	1 (0.2)	214 (35.4)	193 (32)	145 (24.1)	16 (2.6)	32 (5.3)

**Table 3:** Incidence rates (per 1000 live births) for the different perinatal and neonatal outcomes identified, per study site and in total.

Rate (95% CI)	ND	SB	LBW	PTB	SGA	NI				CM
						Tot	BSI	M	RI	
Castellon GUH	2.2 (0.4–6.3)	0.7 (0–4)	113.8 (97.5–131.6)	98.6 (83.5–115.5)	61.2 (49.2–75.1)	6.5 (3–12.3)	5 (2–10.4)	0.7 (0–4)	0.7 (0–4)	13 (7.7–20.4
Dr. Peset UH	0 (0–3.4)	0 (0–3.4)	51.9 (39.5–66.9)	51.9 (39.5–66.9)	55.7 (42.7–71.1)	6.5 (2.6–13.3)	6.5 (2.6–13.3)	0 (0–3.4)	0 (0–3.4)	13 (7.1–21.7)
**Total Valencia**	1.2 (0.3–3.5)	0.4 (0–2.3)	86.7 (75.9–98.6)	78.2 (67.9–89.5)	58.8 (49.8–68.8)	6.5 (3.7–10.5)	5.7 (3.1–9.5)	0.4 (0–2.3)	0.4 (0–2.3)	13 (8.9–18.3)

**Table 4:** Description of the maternal immunization status of all recruited cases in the Valencia Region.

Study Site	Total Number of Mothers; n	Vaccination status; n (%)		
		Vaccinated	Unvaccinated	Unknown
Castellon GUH	161	138 (85.7)	14 (8.7)	9 (5.6)
Dr. Peset UH	86	76 (88.4)	4 (4.7)	6 (7)
**Total Valencia**	247	214 (86.6)	42 (7.3)	15 (6.1)

**Table 5:** Description of maternal immunization of all recruited cases: total number of vaccine doses reported, number of doses for which target disease was known, and the trimester of vaccination.

Study Site	Number of Vaccine Doses Reported; n	Doses for Which Target Disease Was Known; n	Trimester of Vaccination; n (%)			
			First	Second	Third	Unknown
Castellon GUH	193	193 (100%)	9 (4.7)	38 (19.7)	129 (66.8)	17 (8.8)
Dr. Peset UH	97	97 (100%)	2 (2.1)	4 (4.1)	18 (18.6)	73 (75.3)
**Total Valencia**	290	290 (100%)	11 (3.8)	42 (14.5)	147 (50.7)	90 (31)

**Table 6:** Number of total perinatal and neonatal study outcomes recruited for the GAIA definitions applicability assessment during the study period. Number of total cases recruited in the GAIA definitions applicability assessment for each of the outcomes, per site and in total in Valencia Region.

Site	Total Outcomes Identified	Total Outcomes Recruited (% of Identified)	ND; n	SB; n	LBW; n	PTB; n	SGA; n	NI; n	CM; n
Castellon GUH	411	284 (69.1)	3	1	100	104	57	9	11
Dr. Peset UH	193	161 (83.4)	0	0	45	47	48	7	14
**Total Valencia**	604	445 (73.7)	3	1	145	151	105	16	25

**Table 7:** Vaccination status among mothers of recruited cases during pregnancy, and diagnostic certainty level classification among the vaccinated mothers. % represents the percentage among assessed mothers unless explicitly stated.

Site	Mothers Assessed; n	Unknown Vaccination Status; n (%)	UNVACCINATED n (%)	Vaccinated					
				n (%)	Classified n (%)	Not Classified n (%)	Level 1; n (% among Vaccinated Mothers)	Level 2; n (% among Vaccinated Mothers)	Level 3; n (% among Vaccinated Mothers)
Castellón GUH	161	9 (5.6)	14 (8.7)	138 (85.7)	137 (99.3)	1 (0.7)	0 (0)	109 (79.6)	28 (20.4)
Dr. Peset UH	86	6 (7)	4 (4.7)	76 (88.4)	75 (98.7)	1 (1.3)	0 (0)	16 (21.3)	59 (78.7)
**Total Valencia**	247	15 (6.1)	18 (7.3)	214 (86.7)	212 (99.1)	2 (0.9)	0 (0)	125 (59)	87 (41)

## Data Availability

The raw and curated dataset is available through the WHO-GVS-MCC study team, upon request. The data used in the present study is available in the Global Vaccine Safety Multi Country collaboration project measuring risks of early childhood morbid conditions and assessing standardized methods-Final report (Version 1.0, 2020–unpublished, also available upon request to the WHO-GVS-MCC study team).

## Supplementary Materials

The following supporting information can be downloaded at: https://www.mdpi.com/article/10.3390/ijerph19127132/s1, which includes Supplementary Methods, Supplementary Tables S1–S4 and Supplementary Figure S1.
