# Supplementary material for: Risk Measurement of Perinatal and Neonatal Morbidity Characteristics and Applicability of GAIA Case Definitions: Results and Lessons Learnt of a Hospital-Based Prospective Cohort Study in the Valencia Region (2019–2020)
Source: Int J Environ Res Public Health. 2022 Jun 10;19(12):7132. doi: 10.3390/ijerph19127132 (PMC9223180; doi:10.3390/ijerph19127132)
Supplement: Supplementary file 1 [file ijerph-19-07132-s001.zip › ijerph-1729060-supplementary.pdf]

Supplementary Methods

Maternal immunization immunization GAIA levels of diagnostic certainty

Level 1:

- Positive pregnancy test or ultrasound confirmation AND
- Date and time of immunization recorded in medical records by health workers who administered/witnessed vaccination AND
- Details of vaccine administration including lot number, data and time of immunization,

Level 2:

- Confirmed pregnancy by cessation of menstrual period and gravid uterus AND
- Date (at least month and year) of immunization of pregnant woman recorded in medical records who administered/witnessed vaccination AND
- Details of disease against which vaccinated.

Level 3:

- Woman/medical attendant reports pregnancy AND
- Woman reports receipt of vaccination during pregnancy, but no formal recording of immunization available.

Supplementary Tables

Table S1. Classification of the level of diagnostic certainty of the microcephaly cases. Showing % (95% CI).

Site	Recruited cases; n	Any level classified cases; n	% Unclassified
Castellón GUH	11	5	54.5
Dr Peset UH	14	4	71.4
**Total Valencia**	25	9	64

Table S2. Description of the reasons for recruited congenital microcephaly cases not being classifiable to level 3. 1. Information on GA insufficient, n (% of unclassified). 2. HC not 2SD below mean or 3rd percentile according to GA and gender on Intergrowth-21 chart, n (% of unclassified). 3. Not diagnosed based on ICD code algorithm, n (% of unclassified). For each category, the percentage describes the proportion of unclassified recruited cases for which that was the reason why it could not be classified.

Site	N unclassified	1 n (%)	2 n (%)	3 n (%)
Castellón GUH	6	1 (16.7)	5 (83.3)	6 (100)
Dr Peset UH	11	2 (18.2)	10 (90.9)	11 (100)
**Valencia total**	17	3 (17.6)	15 (88.2)	17 (100)

Table S3. Classification of the level of diagnostic certainty of the neonatal infection cases. Showing % (95% CI).

Site	Recruited cases; n	Any level classified cases; n	% classified among recruited
Castellón GUH	8	8	100
Dr Peset UH	7	3	42.9
**Valencia total**	15	11	73.3

Table S4. Maternal immunization: Description of the elements which prevent level 3 maternal immunization ascertainment during pregnancy to be classified as level 1 or 2. The percentage represents the number of cases presenting the element among the level 3 classified cases (n = 87). 1. Immunization not obtained from a primary source medical record, 2. Date of immunization not available, 3. Time of immunization not available, 4. Batch number not available, 5. Disease against which was vaccinated not available, 6. Vaccine brand name not available, 7. Date of immunization (when available) not during pregnancy.

Site	1; n (%)	2; n (%)	3; n (%)	4; n (%)	5; n (%)	6. n (%)	7; n (%)
Castellon GUH	27 (96.4)	2 (7.1)	28 (100)	2 (7.1)	0 (0)	4 (14.3)	1 (3.6)
Dr Peset UH	26 (44.1)	59 (100)	59 (100)	52 (88.1)	0 (0)	58 (98.3)	0 (0)
**Valencia total**	53 (60.9)	61 (70.1)	87 (100)	54 (62)	0 (0)	62 (71.3)	1 (1.1)

Supplementary Figure

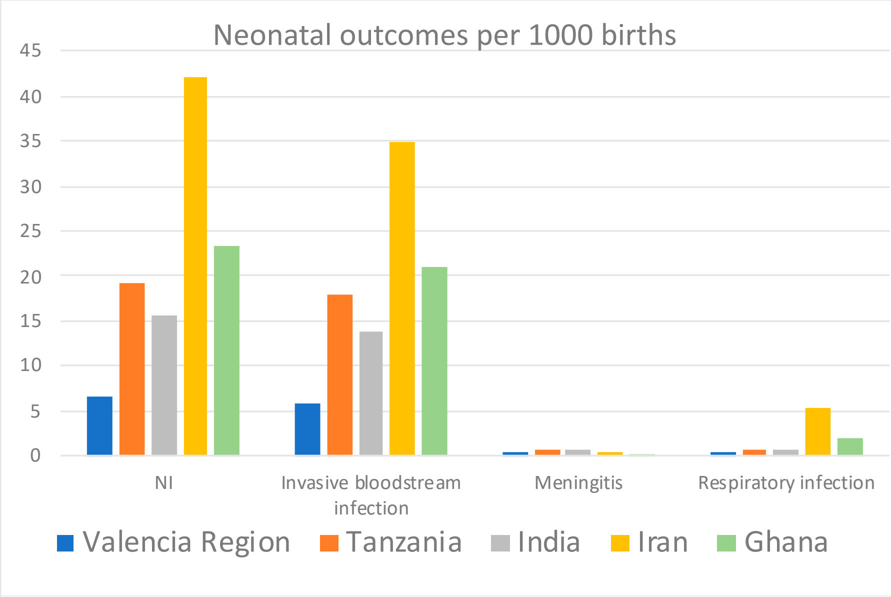

Figure S1. Comparison neonatal infection rates in the Valencia Region, in comparison with the rates in LMIC countries also participating in the WHO-GVS-MCC study. NI: neonatal infections.
